# Measuring Heart Rate Variability Using Commercially Available Devices in Healthy Children: A Validity and Reliability Study

**DOI:** 10.3390/ejihpe10010029

**Published:** 2020-01-10

**Authors:** Kathryn E. Speer, Stuart Semple, Nenad Naumovski, Andrew J. McKune

**Affiliations:** 1 Faculty of Health, Discipline of Sport and Exercise Science/University of Canberra, Canberra (ACT) 2617, Australia; stuart.semple@canberra.edu.au (S.S.); andrew.mckune@canberra.edu.au (A.J.M.); 2 Research Institute for Sport and Exercise/University of Canberra, Canberra (ACT) 2617, Australia; 3 Faculty of Health, University of Canberra, Canberra (ACT) 2617, Australia; nenad.naumovski@canberra.edu.au; 4 Discipline of Biokinetics, Exercise and Leisure Sciences, School of Health Sciences/University of KwaZulu-Natal, Durban 4041, (KwaZulu-Natal), South Africa

**Keywords:** autonomic nervous system or ANS, heart rate variability or HRV, children, photoplethysmography or PPG, Polar H10, validity, reliability

## Abstract

Heart rate variability (HRV) is an accepted method for determining autonomic nervous system activity and cardiovascular risk in various populations. This study assessed the validity and reliability of a commercially available finger photoplethysmography (PPG) system for measuring pediatric HRV in a real-world setting. Sixteen healthy children (4.06 ± 0.58 years) were recruited. The PPG system was compared to the Polar H10 heart rate (HR) sensor validated against ECG (gold standard) for HRV measurement. Seated short-term resting R-R intervals were recorded simultaneously using both systems. Recordings were performed on 3 days at the participants’ school. Paired t-tests, effect sizes and Bland–Altman analyses determined the validity of the PPG system. The relative and absolute reliability of both systems were calculated. No HRV parameters were valid for the PPG system. Polar H10 yielded moderate (0.50–0.75) to good (0.75–0.90) relative reliability with R-R intervals and the standard deviation of instantaneous and continuous R-R variability ratio showing the best results (ICCs = 0.84). Polar H10 displayed better absolute reliability with the root mean square of successive differences, R-R intervals and HR showing the lowest values (TEM% < 12%). The use of the Polar H10 and not the PPG system is encouraged for HRV measurement of young children in an educational real-world setting.

## 1. Introduction

Analysis of heart rate variability (HRV) has provided a non-invasive method for evaluating cardiac autonomic regulation [1]. Predominantly recognized as an independent physiological marker of parasympathetic nervous system (PNS) activity, HRV quantifies the variation in time between consecutive heart beats [2,3]. The time period (in milliseconds) between heart beats is known as the interbeat (R-R) interval and its variation lends clinical insight into disease risk and identification [4]. Under normal resting conditions, healthy individuals exhibit a reciprocating sympathovagal “balance” between the sympathetic nervous system (SNS) and PNS, with the PNS contributing more towards the overall autonomic nervous system (ANS) activity [4,5]. Specifically, ongoing stress relates to poor health such that ANS activity is dysregulated with reduced vagal nerve activity, reflected by a lower HRV [6,7]. In adults, a lower HRV may predict poor psychophysiological health outcomes (e.g., atherosclerosis, diabetes mellitus, major depressive disorder, bipolar disorder) and early mortality [6,8]. A lower HRV may also be associated with harmful lifestyle behaviors (i.e., physical inactivity, poor diet, smoking, stress, etc.) [9].

Regular (i.e., one measurement per day) HRV monitoring is increasingly being applied to the surveillance of clinical, sport and general population health. Many individuals can now assess their daily ANS “status” through a variety of HRV measurement devices, resulting in greater health (e.g., physical and psychological wellbeing) and exercise training optimization [10]. As more commercial HRV measurement devices become available, access for research and medical professionals, coaches, athletes and general individuals to survey ANS health daily becomes feasible with regular HRV monitoring [1,11,12,13]. Furthermore, since seated short-term resting HRV measurements can be reliably achieved in the field (i.e., outside lab-controlled settings), parents and/or teachers now have an increased ability to implement and comply with regular HRV monitoring over time [4,14,15]. 

Investigations into HRV as it relates to health, disease and performance outcomes have been widely conducted in adult populations. From this, HRV testing guidelines for adults have been established and validity and reliability testing of commercially available HRV measurement devices have been conducted [4,16]. However, although HRV has been measured in healthy children, the validity and reliability of commercially available HRV measurement devices for use in young children remains unknown [14]. While the use of an electrocardiogram (ECG) to measure HRV in young children has demonstrated validity and reliability, daily surveillance of HRV using commercially available devices may offer a low-cost convenient method for parents/caregivers to regularly monitor ANS health [4,14,17]. Additionally, the regular monitoring of child ANS health via HRV measurements could enable parents/caregivers to identify early physiological warning signs for highly prevalent diseases, such as cardiovascular disease or depression, thereby correcting or implementing alternative behaviors/activities aimed at improving HRV [18]. Given that young children undergo rapid psychophysiological development and establish lifestyle behaviors that they will likely practice as adults, determining a valid and reliable HRV measurement device for young children in a real-world setting may facilitate a practical daily monitoring method for determining and/or improving ANS health. 

To the authors’ knowledge, commercially available HRV measurement devices have neither been validated nor deemed reliable in young children. Finger monitors have been suggested as an accurate, convenient and quick method to quantify HRV data. As such, this investigation compared a commercially available finger monitor HRV measurement device to a chest strap heart rate (HR) sensor that has been validated against the ECG gold standard [19]. It was hypothesized that the finger monitor would minimize stress and present a safe, easy-to-use method for measuring HRV in a pediatric population. The objective was to compare the validity and reliability of the finger monitor method with the chest strap HR sensor for measuring HRV under normal resting conditions in young children. 

## 2. Materials and Methods 

The study protocol was approved by the University of Canberra Human Research Ethics Committee, Research Ethics and Integrity Review Board (project number 20180384). Prior to participation, the parents of the children provided written and signed informed consent. 

### 2.1. Study Population

Nineteen healthy children (girls: *n* = 7; boys: *n* = 12) between the ages of 3 and 5 years old were recruited from a local early learning center (ELC) located on the University of Canberra campus. Flyers were used for advertising to parents and the recruitment of participants. The director of the ELC emailed the flyers to parents and posted it on the ELC website. Hard copies of informed consent forms and information packets were distributed to the parents of the children attending the ELC. 

Participants were excluded if they had any medical conditions (i.e., autism, ADHD, Asperger’s) or were on any medications (i.e., Adderall, Ritalin, acetylcholinesterase inhibitors or selective serotonin re-uptake inhibitors) that have been shown to impact ANS activity [20,21,22]. Participants were also excluded if the time between consecutive testing sessions exceeded seven days, or participants were sick within the seven days prior to testing. Three girls from the initially recruited 19 participants missed their testing sessions and as a result were excluded. 

### 2.2. Heart Rate Variability Assessment Methods

Each participant was fitted simultaneously with two commercially available HRV devices: (1) the finger monitor and, (2) the Polar H10 HR sensor chest strap. The commercial name of the finger monitor has been kept confidential in accordance with the ethical committee request. The Polar H10 chest strap acted as a reliable comparison for the finger monitor since it has been validated against the ECG gold standard [19]. The Polar H10 chest strap collected and processed HRV measurements by detecting the electrical signals of the heart. The finger monitor used photoplethysmography (PPG) to measure the pulse volume via three multi-wavelength LED emitters, five large visible spectrum photo detectors (wavelength: 565 nm) and one infrared detector (wavelength: 680 nm). With a sampling rate of 500 Hz, the PPG finger monitor used these LED emitters and optical HR detectors to measure blood flow via infrared light shined onto the skin. The photodetectors recorded the variation in light intensities that were transmitted through the skin’s tissue as blood passed through during heart contraction and relaxation. These variations in the peripheral pulse volume represented the R-R intervals [17]. 

Through Bluetooth 4.0 signals, both HRV devices connected wirelessly to the EliteHRV^©^ app downloaded onto two iPads. Once the device connections were confirmed, two “open readings” were commenced simultaneously by pressing the “start” button on the EliteHRV^©^ app. For both devices, signal processing converted either the heart’s electrical signals (extracted from the ECG QRS complex) or the fiducial point in the PPG wave. Notably, the PPG monitor did not generate R-R intervals given that it did not identify the “R-wave” from the signal. Instead, the fiducial point was obtained from the distal (i.e., fingertip) systolic peak in the PPG waveform [17]. The PPG finger monitor calculated the beat-to-beat periods from the measured variation of light intensities absorbed and reflected by red blood cells. The raw R-R interval data were then exported as a text file to Kubios heart rate variability software (version 3.1.0, Biosignal Analysis and Medical Imaging Group, Department of Physics, University of Kuopio, Kuopio, Finland) downloaded onto a Windows 10 laptop (version 1607) for analysis of HRV parameters within the frequency, time and nonlinear domains. Demographic information including age, gender, height and weight was also collected, as these variables were considered influential in past HRV studies (Table 1) [14,23].

Prior to analysis, the R-Rs were manually corrected for ectopic beats using the following guidelines: if an ectopic beat was identified, that beat was deleted and replaced with the average of the two adjacent R-Rs [16,24]. In accordance with the accepted guidelines, all individual participant data included for final analysis did not exceed 20% of the participant’s recording [24]. 

### 2.3. Protocol

The parents of the participants and the ELC staff provided the demographic information required for the study. The testing sessions took place at the ELC between 8:00 and 10:00. Each participant was fitted with the two HRV measurement devices over three testing sessions. The electrodes on the reverse side of the Polar H10 chest strap were moistened with room temperature water prior to being placed on the participant. The Polar H10 chest strap was then fitted around the participant’s chest just below the chest muscles with the HR sensor placed on the xiphoid process of the sternum. Velcro was sewn onto the reverse side of the chest strap so the size could be adjusted for proper fit around the participant. The PPG finger monitor was placed on the participant’s left-hand index finger. Both the Polar H10 chest strap and the PPG finger monitor automatically connected to the Elite HRV^©^ app once a signal was detected.

For each participant, the testing sessions were separated by at least one day. If the time between consecutive testing sessions exceeded seven days, the participant data were discarded. One participant was measured at a time and each HRV recording was 3.5 min long with the first 30 s acting as a stabilization period. The recording time was determined based on recommendations from previous studies [14,25]. 

Prior to HRV recordings, all participants were measured, seated upright with backs pressed up against the back of the same plastic chair. The chair used for testing sessions was familiar to participants as it was borrowed from the ELC. Once the HRV devices were properly placed, participants remained in a resting state for 2 min. For the HRV measurements, the participants were seated and resting in a quiet room while being read a story. The room temperature was kept consistent for all testing days. The purpose of the storybook was to sustain the attention of participants for the HRV measurement duration without compromising the relaxed and quiet state required for measurements. 

### 2.4. Interbeat Interval Analysis 

Using Microsoft Excel 2016, the demographic data [age, height, weight and body mass index (BMI)] was represented as mean ± SD. HRV was determined using the Kubios software program, which analyzed the frequency, time and nonlinear parameters of the manually corrected R-R data. The data were input into Excel using validity and reliability spreadsheets and analyzed for the relationships of Day 2 versus Day 1 and Day 3 versus Day 2 for all HRV parameters [26,27]. The autoregressive (AR) algorithm was used for power spectral analysis of the frequency series and included the log of very low frequency (VLF), low frequency (LF), high frequency (HF) and low frequency of high frequency ratio (LF/HF). The decision to use AR power spectral analysis was based on previous studies’ recommendations for short-term HRV measurements, with the AR algorithm generating better resolution [6,16]. Time and nonlinear parameters were included for investigation, including mean R-R, standard deviation of normal-to-normal intervals (SDNN), mean HR, minimum HR, maximum HR, mean square root differences of the standard deviation (RMSSD), HRV triangular index, triangular interpolation of normal-to-normal interval histogram (TINN), standard deviation of instantaneous R-R interval variability (SD1), standard deviation of continuous long-term R-R variability (SD2) and their ratio (SD1/SD2).

### 2.5. Statistical Analysis

#### 2.5.1. Validity

To determine the validity of the PPG finger monitor, paired sample t-tests were conducted comparing the finger monitor readings with those of the Polar H10 chest strap. Statistical significance was set at *p* < 0.05. Following this, a Pearson correlation between the two methods was performed. However, Pearson correlations only reflect proportional relationships and can therefore lead to misguided interpretation of measures [28]. To establish the agreement between the two HRV measurement devices, Bland–Altman analyses were performed for all HRV parameters [29,30]. This method geometrically illustrates the difference (and limits of agreement) between two clinical measurement devices (i.e., PPG finger monitor vs. Polar H10 chest strap) against each method’s mean [29,31,32]. The graphed data were then analyzed for homoscedasticity and heteroscedasticity. Regarding homoscedasticity, the variability (i.e., error) of one measure is similar to that of the other, whereas heteroscedastic data refers to the disparity between one measure’s variability from the other [32]. Following this, the Hopkins method of interpreting magnitude was used to describe the specific HRV parameter effect sizes: <0.2 trivial; 0.2–0.6 small; 0.6–1.2 moderate, 1.2–2.0 large, 2.0–4.0 very large, >4.0 extremely large [33]. 

#### 2.5.2. Reliability

For the reliability of the two HRV measurement methods, the absolute and relative reliability were calculated with 95% confidence intervals (CI). The absolute reliability was expressed through the typical error of measurement (TEM) and typical error of measurement as a percentage (TEM%). These calculations determined the within-subject variation for each HRV device, indicating the magnitude to which repeated measures varied for participants. TEM represented the actual units of measurement and TEM% denoted a proportion of the mean value [28,34]. The intraclass correlation coefficient (ICC) was calculated to reflect the relative (between-subject variation) reliability [28,34]. ICC calculations were performed instead of Pearson correlations since there were multiple (>2) testing sessions for each participant and the nature of this investigation was univariate [34]. ICC value interpretation followed the guidelines presented by Koo and Li: <0.5 poor; 0.5–0.75 moderate; 0.75–0.9 good; >0.9 excellent [35]. In accordance with the concepts outlined by Hopkins, both absolute and relative reliability and their CIs were included for sufficient interpretation of the HRV measurement methods [30].

## 3. Results

Initially, 19 participants were recruited; however, due to three participants missing their testing sessions, only data from 16 participants (mean ± SD; 4.06 ± 0.58 yrs) were used in the analysis (Table 1). Most participants were male (*n* = 12) with female representation comprising 25% of the total sample. The investigators used the Centre for Disease Control and Prevention (CDC) BMI percentile chart, which takes age- and gender-specific growth patterns into consideration, to interpret healthy vs. unhealthy BMI ranges for children from 2 to 20 years old [36]. According to the CDC BMI percentile chart, interpretation categories are as follows: underweight: <5th percentile; healthy weight: between the 5th and 85th percentile; overweight: >85th percentile, <95th percentile; obese: ≥95th percentile. Regarding the current study, all children were within the healthy weight range, except for three boys who were considered overweight or obese (two overweight, one obese).

### 3.1. Validity of the PPG Finger Monitor

The outcomes of the validity analysis are presented in Table 2. The HF and RMSSD parameters were not normally distributed and were therefore log transformed (Ln) to allow for parametric statistical analysis [37]. Paired sample T-tests revealed significant differences (*p* < 0.05) between the devices for all HRV parameters. Only mean R-R (*p* = 0.25) and mean HR (*p* = 0.19) were not different (not shown). The strongest parameter agreement for the PPG finger monitor compared with the Polar H10 chest strap was the mean HR, with a Pearson correlation of 0.87. The weakest parameter agreement with a Pearson correlation of 0.43 was demonstrated by SDNN (Table 2).

**Figure 1:**
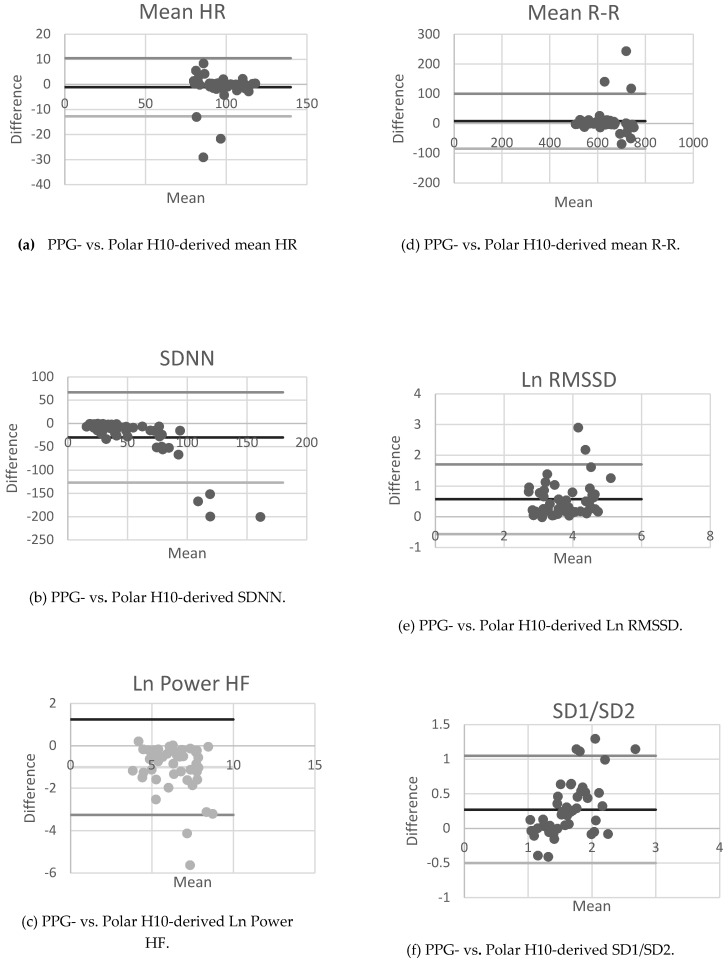
Bland–Altman scatterplots for HRV parameters of healthy children. Note: HRV, heart rate variability; PPG, photoplethysmography; R-R, interbeat intervals; HR, heart rate; SDNN, standard deviation of normal-to-normal intervals; RMSSD, mean square root differences of the standard deviation; HF, high frequency; SD1/SD2, ratio of the standard deviation of instantaneous and continuous R-R interval variability; Ln, natural logarithm.

Bland–Altman plots were also generated for LnHF, LnRMSSD, mean R-R, SDNN, mean HR and SD1/SD2, using pooled data from the three testing sessions (Figure 1). All data indicated homoscedasticity. The Bland–Altman bias with 95% limits of agreement (LOA), 95% CIs and effect sizes are outlined in Table 2. Mean R-R and mean HR indicated the smallest differences between devices, with effects sizes of 0.11 and −0.10, respectively. The largest differences between devices were indicated by LnRMSSD (0.82). The effect sizes demonstrated trivial differences between the two devices for mean R-R and mean HR, whilst the effect sizes were moderate for all HRV parameters (Table 2).

A graphical representation to depict the difference (and limits of agreement) between the two HRV measurement methods (i.e., the PPG finger monitor and the Polar H10 chest strap) against each method’s mean.

### 3.2. Reliability of the PPG Finger Monitor and Polar H10 HR Sensor Chest Strap 

#### 3.2.1. Interclass Correlation 

Polar H10 chest strap ICCs from the first relationship (Day 2 vs. Day 1) ranged from 0.65 (SDNN and LnRMSSD) to 0.76 (mean R-R and mean HR) as compared to an ICC range of 0.12 (SDNN) to 0.65 (mean HR) for the PPG finger monitor. The second relationship (Day 3 vs. Day 2) demonstrated improved ICCs for all the Polar H10 chest strap frequency, time and nonlinear HRV domains, ranging from 0.78 (LnHF) to 0.84 (mean R-R and SD1/SD2). Good reliability was also indicated by SDNN (ICC = 0.82), mean HR (ICC = 0.83) and LnRMSSD (ICC = 0.79). ICCs for the second relationship of the PPG finger monitor ranged from 0.00 (SDNN) to 0.50 (mean HR). However, the PPG finger monitor ICCs for the second relationship demonstrated decreasing values compared with the first. The HRV domains, time and nonlinear measurements displayed greater relative reliability than the frequency measurements. Overall, the PPG finger monitor indicated moderate (0.50–0.75) but mostly poor (<0.50) relative reliability, whilst the Polar H10 chest strap displayed moderate to good (0.75–0.90) relative reliability for the analyzed HRV domains [35] (Table 3). 

#### 3.2.2. Typical Error of Measurement 

Absolute reliability estimates for the HRV domains of the PPG finger monitor compared to the Polar H10 chest strap are presented in Table 3. The Polar H10 chest strap TEM and TEM% demonstrated lower values in the second relationship compared with the first. Contrastingly, the TEM and TEM% values for the PPG finger monitor remained similar across both relationships. Overall, the Polar H10 chest strap indicated lower values for TEM and TEM% than the PPG finger monitor for all HRV parameters, with Ln RMSSD, Ln HF, and SD1/SD2 demonstrating the greatest absolute reliability in terms of TEM and Ln RMSSD, mean R-R and mean HR exhibiting the greatest absolute reliability in relation to TEM%. Comparing the three HRV domains, the time domain appears to be most reliable for within-subject measures in this population. Notably, the nonlinear parameter of SD1/SD2 demonstrated greater reliability for both devices (PPG finger monitor first and second relationship = 17.2% and 16.5%, respectively; Polar H10 chest strap first and second relationship = 18.2% and 14.5%, respectively) in children aged 3–5 years old than its frequency domain counterpart, LF/HF (PPG finger monitor first and second relationship = 65.2% and 46.0%, respectively; Polar H10 chest strap first and second relationship = 65.8% and 45.4%, respectively).

## 4. Discussion

Validity and reliability analyses of PPG HRV measurement have been conducted in a variety of adult populations. However, to the best of our knowledge, the current study is the first to investigate the validity and reliability of a PPG HRV device in children aged 3–5 years old, specifically in a real-world setting. As such, the purpose of this investigation was to determine the validity and reliability of a commercially available PPG system to measure pediatric HRV under normal, resting conditions. The results from the present study suggest that, whilst the PPG finger monitor can produce R-R intervals and HR measurements relatively consistent with the Polar H10 chest strap, all PPG HRV derived recordings are different enough to produce neither valid nor reliable measurements in 3- to 5-year-old healthy children. Moreover, the Polar H10 chest strap appears to provide especially valid and reliable measurements for HRV parameters with strong parasympathetic contribution (e.g., SDNN, LnRMSSD, LnHF and SD1/SD2).

### 4.1. Validity of the PPG Finger Monitor

Pearson correlations and Bland–Altman analyses reflected agreement between R-R intervals and HR and significant discrepancies between the two devices for all HRV parameters (Table 2). Regarding the Pearson correlations, although there was a strong positive relationship between the PPG finger monitor and the Polar H10 chest strap for R-R and HR, only weak to moderate correlations were demonstrated for the HRV parameters. Bland–Altman plots revealed moderate magnitudes of bias and 95% LOA between the PPG finger monitor and the Polar H10 chest strap, with respect to SDNN, LnRMSSD, LnHF and SD1/SD2. Regarding the study population, moderate bias and LOAs could indicate that the differences in the PPG finger monitor compared with the Polar H10 chest strap may be attributed to the PPG device itself rather than study protocol and/or participant characteristics. 

Effect size interpretation indicated trivial differences between the two devices for mean R-R and mean HR. Results revealed significant differences between the PPG finger monitor and the Polar H10 chest strap for all analyzed HRV parameters. This lack of agreement between devices was consistently demonstrated for those HRV parameters with strong PNS contribution, with effect sizes indicating moderate differences for SDNN, LnRMSSD, LnHF and SD1/SD2. 

### 4.2. Reliability of the PPG Finger Monitor

Similarly to the PPG monitor validation analyses, only mean R-R and mean HR demonstrated some, although mostly poor, reliability. Considering the overall between-subject variation for the PPG monitor, none of the parameters (including both HR and HRV measures) indicated good reliability (Table 3). This may suggest that the large degree of variation in repeated measurements for each participant related to the rest of the study sample could be due to mostly false PPG monitor measurements rather than random error. However, it should be noted that this discrepancy could also reflect the study characteristics (i.e., small sample size, similarities between participants) [35]. Regarding the first relationship, mean HR demonstrated the strongest ICC with a moderate relative reliability score for the PPG monitor. Although the ICC worsened for the PPG monitor in the second relationship, it remained the most reliable parameter for between-subject variation. Comparison of the two devices revealed greater relative reliability for all Polar H10 chest strap parameters. Polar H10 chest strap ICCs yielded moderate to good reliability, with mean R-R and SD1/SD2 producing the highest results. As opposed to the PPG finger monitor, the ICCs increased for the Polar H10 chest strap in the second relationship. 

The absolute reliability (TEM%) for the PPG finger monitor and the Polar H10 chest strap followed a consistent pattern as in the relative reliability analysis, with a large degree of within-subject variation being demonstrated in all PPG monitor HRV parameter measures (Table 3). As such, the most reliable measures for the PPG monitor were mean R-R and mean HR, exhibiting the lowest TEM% across both relationships. Mean R-R and mean HR also demonstrated the lowest TEM% for both relationships; however, LnRMSSD, LnHF and SD1/SD2 were also deemed acceptable measurements. This indicates that for each participant, Polar H10 chest strap parameters had a low degree of variation between measurements [28]. Regarding the comparison between the two relationships for the Polar H10 chest strap, both relative and absolute reliability are generally worse for Day 2 vs. Day 1 compared with Day 3 vs. Day 2 (Table 3). This may likely be explained by familiarity. On the first day, participants were less familiar with the investigators, testing procedures and equipment. However, this improved on the second and third testing days. Notably, comparison of the two relationships revealed a lack of improvement in TEM% for parameters measured by the PPG monitor. Moreover, all PPG monitor measurements worsen in the second relationship with respect to ICC. These results suggest that improvement for the Polar H10 chest strap may be attributed to familiarity with error caused by external influences (e.g., momentary disconnection between the participant’s skin and chest strap electrodes), whilst a lack of improvement for the PPG finger monitor is more likely caused by internal software error [28,38,39]. 

This investigation has demonstrated similar findings for valid and reliable HRV measurement via the Polar chest strap in adult populations [1,19,38]. Validity and reliability results from the current investigation for PPG monitor HRV measurements were not consistent with previous study results, which indicated the acceptability of PPG technology [38,39]. It is possible that the discrepancy between the PPG monitor results could be due to the type of PPG system used in the current investigation. Perhaps a forearm, ear or wrist PPG monitor would have proven to be a more valid device for use in 3- to 5-year olds. However, previous studies have indicated caution when interpreting forearm and wrist-worn PPG devices [40,41]. Additional explanations for PPG monitor inconsistencies may be attributed to differences in finger size, position, skin characteristics, room temperature, microcirculation and pressure placed on the sensor (e.g., within the study population or compared to adults) [42,43,44]. These explanations have been generalized to early childhood based on the PPG-derived HRV measurement shortcomings in adult populations. 

### 4.3. Limitations

The current study population was a small convenience sample. Future studies would benefit from recruiting more participants from multiple ELCs for a larger, more representative sample. Secondly, variables known to affect HRV such as lifestyle factors (e.g., diet, physical activity, environment, sleep, etc.) and behavior were not controlled for [9,45]. However, the focus of the study was not to investigate the interaction between these variables and HRV. Instead, the investigators based their inclusion/exclusion criteria on previous research for similar validity and test–retest reliability studies [14]. Also, controlled participant breathing was not enforced in this study due to its questionable influence on HRV and discord within the scientific community, especially at breathing rates <10 breaths/minute [16,46,47]. However, the investigators included analysis of RMSSD, which is less affected by breathing frequency [47,48]. It should also be noted that synchronization of start times for the Polar H10 chest strap and PPG finger monitor may not have been exact despite the investigators best effort to simultaneously press “start” on the two iPads. Moreover, timing lags could have occurred within the EliteHRV© app when switching over from the stabilization to recording period. However, the investigators endeavored to improve and verify the agreement between device signals by matching the timestamps generated by the EliteHRV© app to the timestamps recorded in the “field” setting. Lastly, the seated position of participants may have impacted results due to postural changes and increases in sympathetic activation compared with supine positioning. Indeed, the investigators chose the seated positioning based on practicality and convenience in the participants’ everyday environment, and even though the chair had a back, the presence of postural changes and possible increases in sympathetic activation should be acknowledged [49]. 

### 4.4. Practical Applications

Determining a valid and reliable HRV measurement device for use in young children may establish an effective and easily applicable daily monitoring method for determining ANS health, which may be incorporated into real-world school settings. This daily monitoring method may also help track the response to risk reduction or treatment programs at a stage in life where psychophysiological development is so considerable. Considering the current study, the investigators had to be vigilant of participant finger size with respect to the PPG HRV device so an adequate signal was obtained. Specifically, a signal could be obtained if the participant’s finger was correctly placed; however, the signal was easily lost if the participant’s finger slipped off the sensor or appropriate pressure was not used. Future research should conduct studies investigating the validity and reliability of other PPG monitor in larger, more diverse pediatric populations given its practicality and easy application.

## 5. Conclusions

Regular HRV monitoring via ECG is difficult and impractical for the caregivers of young children. Commercially available HRV devices offer a more practical, cost-effective and easily applicable method for consistently monitoring HRV and ANS “status”. Considering the overall moderate magnitudes of bias, LOAs and effect sizes, poor ICCs as well as relatively higher TEM% for all HRV parameters, the use of the PPG finger monitor in young children is not recommended. The results further suggest that, due to the good ICCs and comparatively lower TEM% alongside the trial improvements, the Polar H10 chest strap appears to be a promising device for recording valid and reliable HRV measurements in 3- to 5-year-old children, especially for those parameters with strong parasympathetic contribution. 

## Figures and Tables

**Table 1:** Descriptive data of male and female healthy participants (Mean ± SD).

	Males (*n* = 12)	Females (*n* = 4)	Significance (*p*)
**Age (years)**	3.95 ± 0.49	4.40 ± 0.77	0.33
**Height (cm)**	103.45 ± 4.28	106.33 ± 5.51	0.47
**Weight (kg)**	17.28 ± 2.48	17.47 ± 2.41	0.91
**BMI (kg/m^2^)**	16.10 ± 1.30	15.39 ± 1.08	0.39

**Table 2:** Indices of validity for heart rate variability parameters of healthy children.

	Chest Strap (mean ± SD)	Finger Monitor (mean ± SD)	Bias (LOA)	Pearson Correlation (95%CI)	Effect Size (Interpretation)
**Mean R-R (ms)**	624.01 ± 75.15	632.10 ± 79.19	8.09 (−84.19 to 100.38)	0.81 (−0.60–0.80)	0.11 (Trivial)
**Mean HR (bpm)**	97.51 ± 11.54	96.35 ± 11.79	−1.15 (−12.77 to 10.46)	0.87 (−0.79–0.59)	−0.10 (Trivial)
**SDNN (ms)**	37.53 ± 18.05	67.50 ± 54.46	−29.98 (−126.67 to 66.72)	0.43 (0.02–1.45)	0.74 (Moderate)
**Ln RMSSD (ms)**	3.51 ± 0.66	4.09 ± 0.75	0.57 (−0.56 to 1.71)	0.67 (0.10–1.54)	0.82 (Moderate)
**Ln Power HF (ms^2^)**	5.84 ± 1.23	6.85 ± 1.53	−1.01 (−3.25 to 1.24)	0.67 (0.01–1.44)	0.73 (Moderate)
**SD1/SD2**	1.75 ± 0.51	1.48 ± 0.30	0.27 (−0.51 to 1.05)	0.63 (−0.65– 0.07)	−0.65 (Moderate)

Note: HRV, heart rate variability; LOA, limits of agreement; CI, confidence interval; R-R, interbeat intervals; HR, heart rate; SDNN, standard deviation of normal-to-normal intervals; RMSSD, mean square root differences of the standard deviation; HF, high frequency; SD1/SD2, ratio of the standard deviation of instantaneous and continuous R-R interval variability; ms, milliseconds; bpm, beats per minute; Ln, natural logarithm.

**Table 3:** Indices of reliability for heart rate variability parameters of healthy children.

HRV Domains		Day 2 vs. Day 1			Day 3 vs. Day 2		
		TEM	TEM (%)	ICC	TEM	TEM (%)	ICC
Mean R-R (ms)	Chest Strap	40.49 (31.13–59.10)	6.7 (5.1–9.9)	0.76 (0.49–0.90)	32.06 (24.65–46.80)	5.3 (4.1–7.9)	0.84 (0.65–0.93)
	Finger Monitor	57.98 (44.91–83.33)	9.0 (6.9–13.1)	0.57 (0.21–0.80)	54.85 (42.49–78.83)	8.9 (6.8–13.1)	0.46 (0.06–0.73)
Mean HR (bpm)	Chest Strap	6.33 (4.87–9.25)	6.7 (5.1–9.9)	0.76 (0.49–0.89)	5.15 (3.96–7.51)	5.3 (4.1–7.9)	0.83 (0.63–0.93)
	Finger Monitor	7.84 (6.07–11.26)	9.0 (6.9–13.1)	0.65 (0.32–0.84)	8.14 (6.30–11.69)	8.9 (6.8–13.1)	0.50 (0.11–0.76)
SDNN (ms)	Chest Strap	10.74 (8.26–15.68)	40.6 (30.0–64.5)	0.65 (0.31–0.84)	8.91 (6.85–13.01)	32.0 (23.8–49.9)	0.82 (0.60–0.92)
	Finger Monitor	53.34 (41.32–76.66)	77.8 (56.2–128.7)	0.12 (−0.30–0.51)	47.03 (36.43–67.59	74.7 (54.0–122.9)	0.00 (−0.42–0.41)
Ln RMSSD (ms)	Chest Strap	0.42 (0.32–0.061)	14.0 (10.6–21.2)	0.65 (0.30–0.84)	0.34 (0.27–0.50)	11.1 (8.5–16.7)	0.79 (0.55–0.91)
	Finger Monitor	0.61 (0.47–0.89)	15.2 (11.5–22.9)	0.41 (−0.03–0.71)	0.57 (0.44–0.83)	14.3 (10.8–21.5)	0.32 (−0.14–0.65)
Ln Power HF (ms^2^)	Chest Strap	0.75 (0.58–1.10)	15.9 (12.0–24.0)	0.68 (0.35–0.86)	0.65 (0.50–0.95)	13.8 (10.5–20.8)	0.78 (0.53–0.91)
	Finger Monitor	1.25 (0.97–1.80)	18.7 (14.2–28.0)	0.41 (0.00–0.71)	1.27 (0.98–1.83)	19.4 (14.8–29.1)	0.21 (−0.23–0.57)
SD1/SD2	Chest Strap	0.30 (0.23–0.43)	18.2 (13.7–27.6)	0.73 (0.44–0.88)	0.23 (0.17–0.33)	14.5 (11.0–21.9)	0.84 (0.65–0.93)
	Finger Monitor	0.24 (0.19–0.34)	17.2 (13.0–25.5)	0.55 (0.17–0.78)	0.23 (0.18–0.33)	16.5 (12.6–24.6)	0.44 (0.03–0.72)

Note: HRV, heart rate variability; TEM, typical error of measurement (90% confidence interval); TEM%, typical error of measurement as a percentage (90% confidence interval), ICC, intraclass correlation coefficient (95% confidence interval); R-R, interbeat intervals; HR, heart rate; SDNN, standard deviation of normal-to-normal intervals; RMSSD, mean square root differences of the standard deviation; HF, high frequency; SD1/SD2, ratio of the standard deviation of instantaneous and continuous R-R interval variability; ms, milliseconds; bpm, beats per minute; Ln, natural logarithm.
